# Effects of Endourologic Devices and Connectors on Irrigation Flow Through the Flexible Ureteroscope Working Channel

**DOI:** 10.1155/aiu/7192796

**Published:** 2026-07-30

**Authors:** Oleksandr Chepeliuk, Rinat Lasmanovich, Isaias Caballero Lopez, Dina Orkin, Miki Haifler, Dorit E. Zilberman, Zohar A. Dotan, Asaf Shvero, Nir Kleinmann

**Affiliations:** ^1^ Department of Urology, Sheba Medical Center, Tel HaShomer, Ramat Gan, Israel, sheba.co.il; ^2^ Faculty of Medicine, Tel Aviv University, Tel Aviv, Israel, tau.ac.il; ^3^ Operative Room Division, Anesthesia and Pain, Sheba Medical Center, Tel HaShomer, Ramat Gan, Israel, sheba.co.il; ^4^ Meir Medical Center, Kfar Saba, Israel, mmc.org.il

**Keywords:** connector systems, flexible ureteroscopy, irrigation flow, laser fibers, ureteral access sheath

## Abstract

**Purpose:**

To evaluate the effect of different connector types and commonly used endourologic devices on irrigation fluid flow through the working channel of a flexible ureteroscope.

**Methods:**

Irrigation flow was measured under a 200‐cm H_2_O pressure head using three connector types (Tuohy‐Borst, UroLok, and Check‐Flo) and a standard Y‐set connection. Instruments tested included Piranha biopsy forceps, Segura hemisphere basket, Glidewire guidewire, and two laser fibers (200 μm and 365 μm). Each condition was repeated six times. Descriptive statistics were calculated, and one‐way ANOVA was used to assess differences between groups.

**Results:**

With an empty channel, the standard Y‐set connection achieved the highest mean flow (115.8 ± 4.3 mL/min), followed by Tuohy‐Borst (113.0 ± 1.6), UroLok (100.2 ± 1.3), and Check‐Flo (80.4 ± 0.5) (*p* < 0.001). Inserting instruments markedly reduced flow. The Piranha forceps and Glidewire guidewire produced the largest reduction (∼96–97%). A larger instrument diameter or the absence of a smooth coating significantly reduced flow across all connectors. With small‐caliber instruments, connector design played a greater role: relative flow reduction was greatest with Tuohy‐Borst (50.6%) and lowest with Check‐Flo (38.0%). In absolute terms, UroLok yielded the highest median flow with laser fibers (38.7 mL/min), followed by Tuohy‐Borst (37.5 mL/min) and Check‐Flo (34.7 mL/min).

**Conclusion:**

Both connector choice and instrument size significantly affect irrigation flow in flexible ureteroscopy. Large‐diameter instruments almost abolish flow regardless of connector, whereas with smaller instruments, connector design influences performance. These findings emphasize the importance of selecting optimal connector–instrument combinations to maintain intraoperative visibility.

## 1. Introduction

During retrograde intrarenal surgery, auxiliary instruments such as guidewires, laser fibers, biopsy forceps, and baskets are passed through the ureteroscope’s working channel. Despite advances in the miniaturization of scopes and endourologic devices, two major challenges persist when using these devices through a ureteroscope: (1) limited tip deflection, restricting access to certain calyces, and (2) reduced irrigation flow through the working channel, which compromises intrarenal visibility.

In gravity‐driven irrigation systems, further miniaturization of the working channel has only exacerbated the problem of limited irrigation flow and suboptimal visualization. Modern digital flexible ureteroscopes typically contain a single working channel that must simultaneously provide irrigation and allow passage of instruments [1–3]. Pressurized irrigation systems, which force fluid into the working channel under increased pressure, have improved intraoperative fluid clearance and partially overcome the limitations imposed by small channel diameters. At the same time, the introduction of ureteral access sheaths (UASs) has enhanced fluid outflow, improving visibility through more effective washout of blood, stone dust, and debris. Additional benefits of UAS include cooling of the operative field, allowing for the safe use of higher‐power lasers, and reducing intrarenal pressure (IRP) and backflow [4].

Several factors influence irrigation flow: the type of fluid delivery system (gravity vs. pressurized), the inner diameter of the working channel, the caliber and coating of the instrument, and the capacity for washout and fluid egress [5]. Multiple experimental studies have investigated these variables, including irrigation bag height, irrigation pressure, working channel diameter, tip deflection, instrument caliber, and their effects on irrigation flow and visibility [6–8].

Instrument caliber has consistently been shown to significantly affect irrigation flow. Higher irrigation pressure increases flow, but in the presence of a working instrument, flow decreases proportionally with the instrument’s working channel cross‐sectional area [9]. The presence of instruments may reduce flow by up to 95%, while deflection alone may reduce it by as much as 50% [6, 10]. Interestingly, some studies suggest that scope deflection alone has minimal effect on flow metrics [11].

Beyond irrigation inflow, adequate outflow is equally important. The development of different models of UAS has improved washout, visibility, IRP control, and irrigation fluid temperature, thereby lowering the risks of backflow and bacteremia [12]. Moreover, inserting an auxiliary open‐ended ureteral catheter alongside a flexible ureteroscope with an instrument in the working channel has been shown to increase irrigation flow to the surgical field by up to sevenfold [4].

Irrigation fluid flow is therefore a critical determinant of visualization, irrigation fluid temperature control, and removal of blood, epithelial debris, and stone fragments during flexible ureteroscopy. Our hypothesis is that the choice of connector at the proximal end of the ureteroscope, combined with the type of endourologic instrument in the working channel, may significantly influence irrigation dynamics.

## 2. Materials and Methods

In this in vitro study, we performed two experimental steps using a single‐use 7.5‐Fr digital flexible ureteroscope (Innovex Medical Co., Ltd., Shanghai, China) with a 3.6‐Fr working channel. Irrigation was provided via a standard Y‐set connected to a 3‐L saline bag under constant hydrostatic pressure of 200 cm H_2_O delivered by a mechanical pump. This starting pressure was chosen as it represents our standard clinical practice for procedures utilizing UAS. The ureteroscope was laid flat on a table with the same distance from the irrigation pump and the collection container. Effluent was collected in a graduated cylinder, and the volume over 1 min was recorded. *Step 1:* Baseline flow with connectors: Irrigation flow was measured under four conditions: direct connection of the Y‐set to the ureteroscope (no connector, serving as the gold standard for maximal achievable flow), Tuohy‐Borst adapter (Cook Medical, Bloomington, IN, USA), UroLok adapter (Boston Scientific, Marlborough, MA, USA), and Check‐Flo valve (Cook Medical). These connectors are available in our institution and are commonly used. Each connector setup measurement was repeated six times, for a total of 24 rounds. *Step 2*: Flow with endourologic devices: Five commonly used instruments were tested with each connector: 3‐Fr Piranha biopsy forceps (Boston Scientific), Segura hemisphere stone basket (∼2.4 Fr) (Boston Scientific), Glidewire guidewire (0.038″) (Terumo Medical Corporation, Somerset, NJ, USA), holmium laser fiber (Boston Scientific) SlimLine 200 (core diameter: 272 μm; maximal outer diameter of 450 μm), and holmium laser fiber (Boston Scientific) SlimLine 365 (core diameter: 365 μm; maximal outer diameter of 580 μm). Each device was introduced so that approximately 1 cm protruded beyond the distal end of the ureteroscope (thus passing through its entire length). Irrigation was measured as in Step 1. For each instrument–connector combination, six repetitions were performed (5 instruments × 3 connectors × 6 = 90 rounds). Before each test, the ureteroscope was flushed with 50‐cc saline.

Statistical analysis: Mean ± standard deviation and range were used to summarize flow rates (mL/sec). One‐way ANOVA test was applied to assess differences between connectors and between instrument conditions. A *p* value< 0.05 was considered statistically significant.

## 3. Results

### 3.1. Step 1

With an empty working channel, the highest mean irrigation flow was recorded when the Y‐set was connected directly to the ureteroscope without a connector (115.8 ± 4.3 mL/min), representing the maximal achievable flow at 200 cm H_2_O pressure. The addition of connectors reduced flow to varying degrees (Table 1).

**TABLE 1:** Mean irrigation fluid flow rate.

Connector type	Mean (mL/min)	Standard deviation (±)	Range (mL/min)
Without connector	115.8	4.3	112–123
Tuohy‐Borst	113	1.6	111–115
UroLok	100.2	1.3	99–102
Check‐Flo	80.4	0.5	80–81

*Note:* mL: milliliter; min: minute. Mean irrigation fluid flow rate, standard deviation, and range with straight connection of Y‐set compared to 3 connector types: Tuohy‐Borst, UroLok, and Check‐Flo.

**FIGURE 1:**
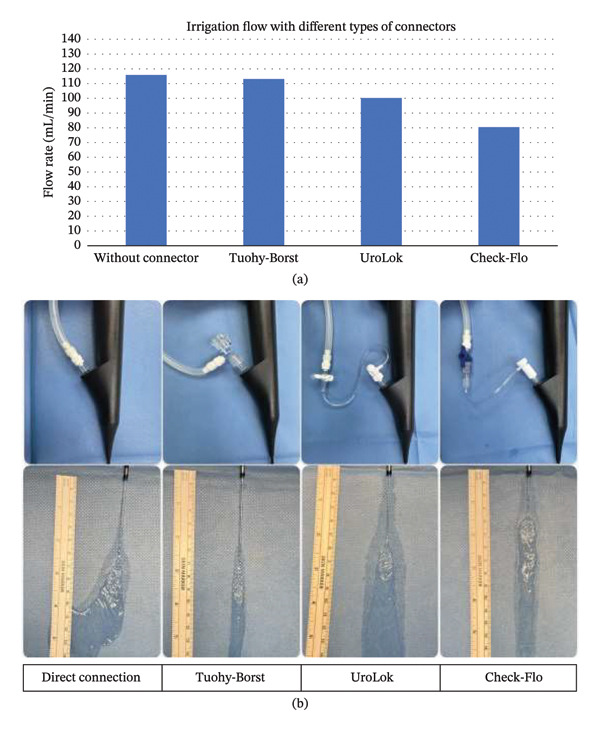
Irrigation flow with and without connectors and their jet visualization. cm: centimeter. (a) Mean irrigation fluid flow rate and standard deviation through the working channel of a flexible ureteroscope with connection of Y‐set compared to 3 connector types: Tuohy‐Borst, UroLok, and Check‐Flo. (b) Visualization of irrigation fluid jet of a flexible ureteroscope with straight connection of Y‐set compared to 3 connector types—Tuohy‐Borst, UroLok, and Check‐Flo—used in the experiment. Connector tubing was bent for illustration purposes. To visualize the flow, the ureteroscope was laid flat directly against a standard blue surgical drape. The visible “fluid jet” represents the wetting pattern on the drape, serving as a two‐dimensional representation of the irrigation stream’s dispersion and reach.

One‐way ANOVA confirmed significant differences between connector types (*p* < 0.001). Flow rates ranked highest with direct connection, followed by Tuohy‐Borst, UroLok, and Check‐Flo (Figure 1). The Check‐Flo valve demonstrated the most consistent performance across repetitions, with the lowest variability (SD = 0.5 mL/min).

### 3.2. Step 2

#### 3.2.1. Piranha Biopsy Forceps (3‐Fr Instrument)

**FIGURE 2:**
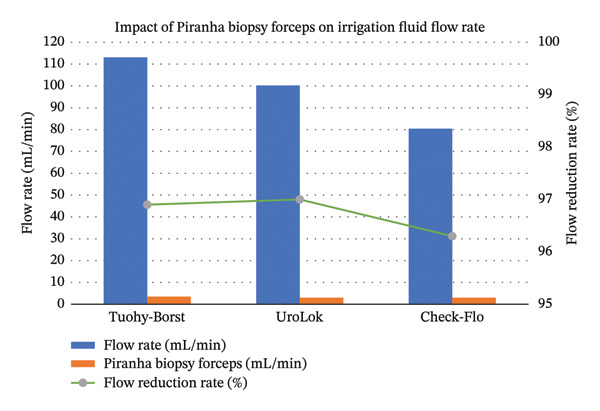
Impact of Piranha biopsy forceps on irrigation flow rates. mL: milliliter; min: minute. Impact of Piranha biopsy forceps introduced into the working channel of the flexible ureteroscope on irrigation fluid flow rate while using different connectors.

We next evaluated the effect of inserting the Piranha biopsy forceps (diameter: 1.0 mm, ∼3 Fr) into the working channel via each of the three connector types. This instrument occupies approximately 83% of the 3.6‐Fr channel lumen, resulting in a substantial obstruction of irrigation flow. Being an uncoated instrument, we also observed minor backflow of irrigation fluid through the connectors during testing. Across all connectors, the relative impact was similar: insertion of the Piranha forceps reduced irrigation flow by approximately 96%–97% compared with baseline (Figure 2). Given the near‐complete loss of flow, differences between connector types became negligible. Thus, when using large instruments such as the Piranha forceps, irrigation flow is severely restricted regardless of connector choice, and the connector type plays little practical role.

#### 3.2.2. Segura Stone Basket (∼2.4‐Fr Instrument)

**FIGURE 3:**
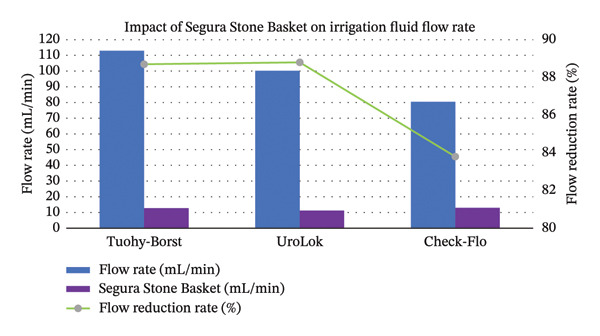
Impact of Segura biopsy basket. mL: milliliter; min: minute. Impact of Segura biopsy basket introduced into the working channel of the flexible ureteroscope on irrigation fluid flow rate while using different connectors.

We next assessed the impact of the Segura biopsy basket (diameter ∼0.8 mm, ∼2.4 Fr) on irrigation flow. Insertion of the basket significantly reduced flow across all connectors. Among them, the Check‐Flo valve maintained the highest mean flow (13 mL/min), followed closely by the Tuohy‐Borst adapter (12.8 mL/min), while the UroLok adapter yielded the lowest flow (11.2 mL/min). The differences in absolute values between connectors were small, on the order of 1–2 mL/min, and thus clinically negligible (Figure 3). Overall, all connectors demonstrated a marked reduction in flow with the Segura basket in situ, with relative reductions consistent across connectors, differing by only ∼5% points.

#### 3.2.3. Laser 200 Micron

**FIGURE 4:**
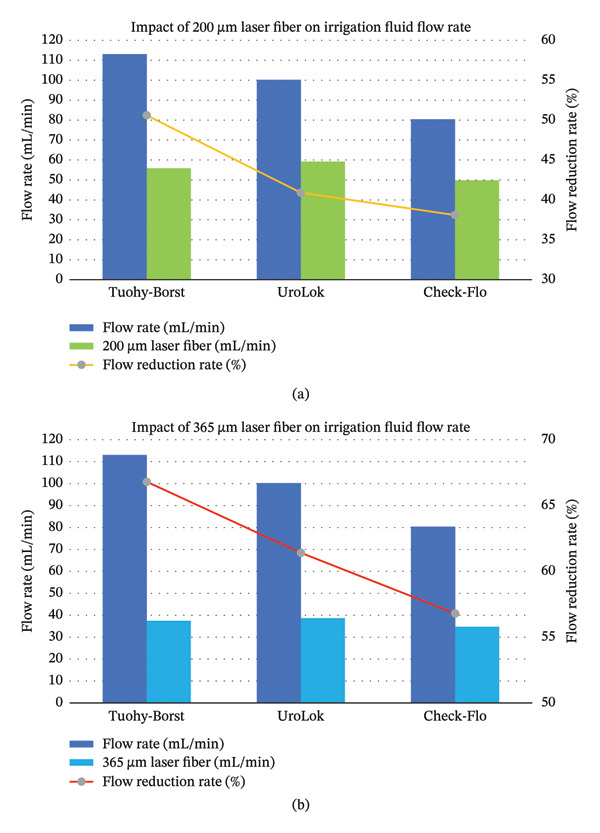
Flow rates and reduction percentages with laser 200 microns and 365 microns. mL: milliliter; min: minute. Impact of laser 200 microns (a) and 365 microns (b) introduced into the working channel of the flexible ureteroscope on irrigation fluid flow rate while using different connectors.

When a 200‐μm laser fiber was inserted, mean irrigation flow rates remained higher than with larger instruments but still showed notable reductions compared with baseline. The UroLok adapter provided the highest mean flow (59.2 mL/min), while the Check‐Flo valve yielded the lowest (49.8 mL/min). The Tuohy‐Borst adapter produced an intermediate value. In terms of relative reduction from baseline, Tuohy‐Borst showed the greatest decrease (50.6%), whereas Check‐Flo had the smallest (38.0%). The UroLok connector demonstrated a moderate reduction (40.9%) (Figure 4a). These findings indicate that with small‐caliber instruments such as the 200‐μm fiber, connector design plays a more prominent role in irrigation flow compared to when larger instruments are used.

#### 3.2.4. Laser 365 Micron

When a 365‐μm laser fiber was introduced, irrigation flow decreased further compared with the thinner 200‐μm fiber, as expected due to the larger instrument diameter occupying more of the working channel. The trend among connectors, however, remained similar. The UroLok adapter provided the highest median flow (38.7 mL/min), followed by the Tuohy‐Borst adapter (37.5 mL/min), while the Check‐Flo valve yielded the lowest flow (34.7 mL/min) (Figure 4b). These findings confirm that larger instrument diameter results in greater flow reduction, but relative differences between connectors are preserved, with UroLok consistently maintaining slightly higher flow rates than Tuohy‐Borst and Check‐Flo.

#### 3.2.5. General Results

**FIGURE 5:**
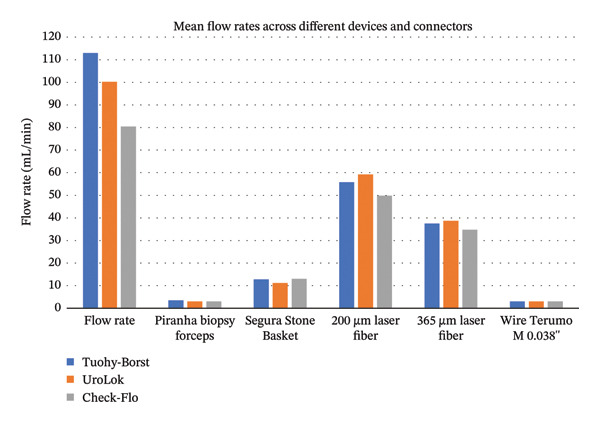
Impact of different working endourological instruments on irrigation fluid flow rate. Impact of different endourological working instruments, introduced through the flexible ureteroscope’s working channel, on irrigation flow rate. *X*‐axis: instrument type; *Y*‐axis: irrigation flow rate (mL\min). Connector types are shown in the upper‐right inset.

With small‐caliber instruments such as laser fibers, the UroLok adapter consistently maintained slightly higher flow rates than the other connectors, although the differences were small and unlikely to be clinically meaningful. The highest absolute flow was observed with the Tuohy‐Borst adapter when the working channel was empty. As instrument diameter increased, or when instruments lacked a smooth surface coating, flow rates declined significantly across all connectors. This confirms that instrument size and characteristics are the dominant determinants of flow, whereas connector type plays a secondary role, becoming more relevant only when the channel is occupied by small‐diameter instruments. The impact of different endourological instruments on irrigation fluid flow rate is demonstrated in Figure 5.

## 4. Discussion

### 4.1. Principal Findings and Clinical Implementation

Optimal visibility during retrograde intrarenal surgery depends on maintaining the highest possible irrigation flow, which ensures rapid field renewal and effective washout of blood and debris. In our study, the straight connection of the irrigation Y‐set to the ureteroscope yielded the highest flow, representing the maximal achievable irrigation rate. This approach may be useful during diagnostic ureterorenoscopy, when no instruments are inserted, and maximal visualization is required to identify pathology. However, it precludes subsequent introduction of instruments, making it impractical for therapeutic steps.

The Tuohy‐Borst connector reduced flow by only 2.8 mL/min (2.4%) compared to the straight connection, suggesting it can be safely used from the start of the procedure without significantly compromising visibility. This allows uninterrupted introduction of instruments and avoids repositioning or manipulation of the scope during connector exchanges.

Insertion of large‐diameter instruments such as the Piranha biopsy forceps (1 mm, 3 Fr) drastically reduced flow (∼96–97%), irrespective of connector type. The absence of a coating also contributed to fluid backflow at the connector. Similarly, the Glidewire guidewire produced a comparable reduction. These findings highlight that instrument size and surface properties dominate irrigation dynamics, overshadowing the influence of connector design.

In contrast, smaller instruments such as laser fibers cause less dramatic reductions. The 200‐μm laser reduced flow by ∼39–51%, whereas the 365‐μm laser (occupying 30.5% of the channel cross section) reduced flow by ∼58–67%. These results are consistent with prior studies, such as Nagele et al., who reported a twofold drop in flow when switching from 1.5‐Fr to 1.9‐Fr baskets [5]. The UroLok adapter maintained slightly higher absolute flow rates with thin instruments, although the difference compared with other connectors was modest (5%–10%) and may not be clinically significant.

Connectors vary in their internal geometry, lumen diameter, valve design, and length, creating varying degrees of proximal dead space and hydraulic resistance [13]. When instruments of different calibers are inserted, the effective residual lumen available for irrigation becomes the annular space between the instrument and the channel wall. In this situation, even small differences in connector internal diameter, sealing mechanism, or valve configuration may significantly influence resistance. Large‐caliber or uncoated instruments not only reduce the available cross‐sectional area but also may disrupt laminar flow, increase turbulence, and further reduce effective irrigation. These hydrodynamic considerations may partially explain the observed differences between connectors when smaller instruments such as laser fibers were used, while becoming negligible when large instruments nearly occlude the channel [13].

From a device design perspective, irrigation capacity is not only determined by connectors and instruments but also by scope characteristics. Factors such as working channel diameter, field of view, and degree of deflection affect both flow and maneuverability. Dual‐channel ureteroscopes have been proposed to overcome these limitations, enabling simultaneous irrigation and instrumentation. Some studies have reported up to a 37‐fold increase in irrigation flow compared with single‐channel scopes under specific conditions, although baseline flow in dual‐channel scopes may be lower (≈90 mL/min) than that in single‐channel scopes without an instrument (115 mL/min in our study) [2]. Clinically, the effect of dual channels can be mimicked by placing an open‐ended ureteral catheter alongside the ureteroscope through a UAS or by using specialized UAS with integrated irrigation channels or dual‐diameter working channel [14, 15].

When large instruments severely restrict flow, increasing irrigation pressure with compressor or pump devices can improve visibility [16]. While lower pressures (e.g., 40 mmHg) are often recommended for sheathless procedures to mitigate elevated IRP, the 200‐cm H_2_O starting pressure used in our model reflects standard practice when a UAS is employed. The sheath facilitates continuous outflow, maintaining safe IRP [4, 12]. Furthermore, the high pressure generated at the pump is substantially attenuated at the distal tip of the scope whenever an instrument occupies the working channel [8, 11]. However, this must be balanced against the risk of elevated IRP, emphasizing the importance of outflow via UAS or parallel drainage.

Beyond mechanical pressure attenuation, the physiological impact of IRP must be carefully managed. Sustained IRP elevation above 30–40 mmHg, often caused by high irrigation flow without adequate outflow, drives pyelovenous backflow and significantly increases the risk of postoperative SIRS and sepsis. While conventional UAS provides passive fluid exit, recent literature highlights a shift toward active pressure management using suction ureteral access sheaths (S‐UASs). By applying continuous negative pressure, S‐UAS actively prevents dangerous IRP spikes and accelerates stone clearance even under maximal pump settings. Consequently, understanding the specific fluid dynamics of different connectors and instruments is essential to balance inflow with the chosen outflow strategy, ensuring procedural safety [17, 18].

### 4.2. Limitations

Our study has several limitations. First, it was conducted in an in vitro open‐drainage system, simulating conditions where inflow equals outflow. In vivo, IRP and variable outflow resistance may alter flow dynamics, making direct translation imperfect. Second, only one model of single‐use digital ureteroscope was tested. While absolute flow rates may vary between devices, the trends observed should be broadly applicable. Third, the Tuohy‐Borst connector showed some variability due to manual tightening of its locking mechanism, which occasionally caused retrograde leakage around instruments. By contrast, the UroLok and Check‐Flo connectors provided more consistent sealing. Finally, we did not directly evaluate visibility outcomes (clarity of the operative field) but used flow as a surrogate parameter.

## 5. Conclusion

The choice of connector and the type of instrument used both influence irrigation flow during flexible ureteroscopy. Large‐diameter or uncoated instruments remain the dominant limiting factor, markedly reducing flow regardless of connector. When small‐caliber devices are used, connector design becomes more relevant, though differences in absolute flow rates are modest. Clinicians should balance the trade‐off between maximizing irrigation flow and ensuring stable performance when selecting connector–instrument combinations to optimize intraoperative visibility.

## Author Contributions

Oleksandr Chepeliuk: conceptualization, methodology, investigation, data curation, formal analysis, writing–original draft, visualization, and corresponding author.

Rinat Lasmanovich: investigation, data curation, and writing–review and editing.

Isaias Caballero Lopez: investigation, data curation, and writing–review and editing.

Dina Orkin: methodology, supervision, and writing–review and editing.

Miki Haifler: formal analysis, visualization, and writing–review and editing.

Dorit E. Zilberman: supervision and writing–review and editing.

Zohar A. Dotan: supervision, project administration, and writing–review and editing.

Asaf Shvero: conceptualization, supervision, and writing–review and editing.

Nir Kleinmann: conceptualization, methodology, supervision, project administration, and writing–review and editing.

## Funding

This research received no specific grant from any funding agency in the public, commercial, or not‐for‐profit sectors.

## Disclosure

All authors reviewed and approved the final version of the manuscript.

## Conflicts of Interest

The authors declare no conflicts of interest.

## Data Availability

The data supporting the findings of this study are available from the corresponding author upon reasonable request.
